# STI prevalence among men living with HIV engaged in safer conception care in rural, southwestern Uganda

**DOI:** 10.1371/journal.pone.0246629

**Published:** 2021-03-03

**Authors:** Pooja Chitneni, Mwebesa Bosco Bwana, Winnie Muyindike, Moran Owembabazi, Paul Kato Kalyebara, Adolf Byamukama, Yona Mbalibulha, Patricia M. Smith, Katherine K. Hsu, Jessica E. Haberer, Angela Kaida, Lynn T. Matthews

**Affiliations:** 1 Division of Infectious Diseases, Massachusetts General Hospital, Boston, MA, United States of America; 2 Division of Infectious Diseases and General Internal Medicine, Brigham and Women’s Hospital, Boston, MA, United States of America; 3 Global Health Collaborative, Mbarara, Uganda; 4 Mbarara Regional Referral Hospital and Mbarara University of Science and Technology, Mbarara, Uganda; 5 Epicentre, Médecins Sans Frontières (MSF), Mbarara, Uganda; 6 Faculty of Health Sciences, Simon Fraser University, Burnaby, British Columbia, Canada; 7 Division of STD Prevention, Massachusetts Department of Public Health, Boston, MA, United States of America; 8 Section of Pediatric Infectious Disease, Boston University Medical Center, Boston, MA, United States of America; 9 Center for Global Health, Massachusetts General Hospital, Boston, MA, United States of America; 10 Department of Medicine, Harvard Medical School, Boston, MA, United States of America; 11 Division of Infectious Diseases, University of Alabama at Birmingham, Birmingham, AL, United States of America; Nagoya University Asian Satellite Campuses Institute, Nagoya University Graduate School of Medicine, MYANMAR

## Abstract

HIV care provides an opportunity to integrate comprehensive sexual and reproductive healthcare, including sexually transmitted infection (STI) management. We describe STI prevalence and correlates among men living with HIV (MLWH) accessing safer conception care to conceive a child with an HIV-uninfected partner while minimizing HIV transmission risks. This study reflects an ongoing safer conception program embedded within a regional referral hospital HIV clinic in southwestern Uganda. We enrolled MLWH, planning for pregnancy with an HIV-uninfected partner and accessing safer conception care. Participants completed interviewer-administered questionnaires detailing socio-demographics, gender dynamics, and sexual history. Participants also completed STI laboratory screening for syphilis (immunochromatographic testing confirmed by rapid plasma reagin), and chlamydia, gonorrhea, trichomoniasis, and HIV-RNA via GeneXpert nucleic acid amplification testing. Bivariable associations of STI covariates were assessed using Fisher’s exact test. Among the 50 men who completed STI screening, median age was 33 (IQR 31–37) years, 13/50 (26%) had ≥2 sexual partners in the prior three months, and 46/50 (92%) had HIV-RNA <400 copies/mL. Overall, 11/50 (22%) had STIs: 16% active syphilis, 6% chlamydia. All participants initiated STI treatment. STI prevalence was associated with the use of threats/intimidation to coerce partners into sex (27% vs 3%; p = 0.03), although absolute numbers were small. We describe a 22% curable STI prevalence among a priority population at higher risk for transmission to partners and neonates. STI screening and treatment as a part of comprehensive sexual and reproductive healthcare should be integrated into HIV care to maximize the health of men, women, and children.

## Introduction

Globally in 2016 there were 376 million new cases of the four most prevalent, curable STIs: syphilis, chlamydia, gonorrhea, and trichomoniasis [1]. Untreated maternal STIs pose a risk to women and also to neonates through preterm birth, disability, and stillbirth [2]. Laboratory-based STI screening is limited in lower-resourced settings, and available tests typically focus on pregnant women to prevent perinatal STI transmission.

STI care is a critical component of sexual and reproductive health (SRH). Individuals planning for pregnancy are uniquely vulnerable to STI and HIV transmission as most will engage in condomless sex to conceive a child [3, 4]. Such risks are amplified in settings like Uganda with high fertility rates (5.1 births/woman [5]), high HIV prevalence (7% adult HIV prevalence [6]), and a lack of routine laboratory STI screening. For example, among women in Uganda who reported wanting a child and vulnerable to HIV acquisition, 24% had a curable STI [7]. Screening, diagnosing, and treating STIs among women and men prior to pregnancy can prevent infections like syphilis, which can traverse the placenta and cause in-utero and post-natal morbidity [8].

Diagnosing and treating men is crucial to bolstering men’s health and to preventing STI recurrence and facilitating cure among women and neonates. Safer conception programming educates and equips HIV-affected individuals and couples with the tools needed to prevent HIV transmission and optimize health while safely conceiving a child [9–11]. These programs are ideal settings for laboratory-based STI screening. However, SRH research is currently limited among men planning for conception, and STIs in this population are rarely described.

The objective of this study was to estimate the prevalence and correlates of four curable STIs (syphilis, chlamydia, gonorrhea, and trichomoniasis) among a cohort of men living with HIV (MLWH) in rural, southwestern Uganda, engaged in a safer conception care program, and seeking pregnancy with an HIV sero-negative or serostatus-unknown partner.

## Materials and methods

### Study setting

This study took place among clients attending the Healthy Families Clinical Pilot Program integrated within the Mbarara Regional Referral Hospital HIV clinic. Since December 2016, this program has provided safer conception counseling and care to over 375 serodifferent couples [12, 13]. Safer conception programming encompasses strategies to reduce HIV transmission during the process of achieving pregnancy, and includes ART-mediated viral suppression, couples-based HIV counseling and testing, counselling to delay condomless sex until HIV-RNA suppression, timing condomless sex to peak fertility, and where available, providing access to daily, oral, tenofovir-based pre-exposure prophylaxis (PrEP) for uninfected partners.

### Study design and participants

The “Getting to Zero” study was a six-month, mixed-methods, pilot, prospective cohort study assessing safer conception care uptake among a cohort of 50 MLWH, who expressed personal or partner plans to have a child. Inclusion criteria entailed being an adult male (aged 18+) living with HIV, in a partnership with an HIV sero-negative or serostatus-unknown female partner (per male participant report), planning to conceive a child in the next year, and naïve to the Healthy Families Clinical Program. Participants were recruited from the HIV clinic and through community outreach from October 2018 to March 2019.

### Study procedures

Participants attended study visits at enrollment, three-, and six-months which entailed individual safer conception counseling [9]. Participants were encouraged to bring desired, female, pregnancy-partners to subsequent study visits for engagement in care. Safer conception counseling included treatment as prevention, PrEP for HIV-uninfected partners, timing condomless sex to peak fertility, contraception use until ready for conception, sperm washing and insemination, and treating STIs [13].

### Questionnaire

Participants completed interviewer-administered questionnaires at enrollment and six-months in English or Runyankole, the primary local language. The questionnaires encompassed information on socio-demographics, alcohol use [14], sexual and reproductive behaviors, HIV medical history, desired pregnancy-partner HIV disclosure and acquisition risk perception, intimate and sexual partner violence [15], the Sexual and Relationship Power Scale [16, 17], the Dyadic Trust Scale [18], and the Duke-University of North Carolina Social Support Questionnaire [19]. These covariates were chosen due to their association with STI in prior studies [20, 21].

The questionnaire items were collected using tools refined by our research team in prior research studies with men living with HIV [22] and women living without HIV and considering PrEP in Uganda [7]. The AUDIT scale measures alcohol use and has been validated in six countries including Nigeria [14, 23]. The Sexual and Relationship Power Scale measures relationship control and decision-making dominance and was originally validated in the United States. We used two forms of the Sexual and Relationship Power Scale: the eight-item Sexual and Relationship Power Decision-Making Dominance subscale [16] and the 13-item Sexual and Relationship Power Equity scale which was adapted and validated in the South African context [17]. The Dyadic Trust Scale is an 8-item scale that measures trust and benevolence among sexual partners and was validated in the United States [18]. The Duke-University of North Carolina Social Support Questionnaire measures trust and benevolence among sexual partners and has been validated in the United States and Rwanda [19, 24]. We utilized 10 of the 14 Duke-University of North Carolina Social Support Questionnaire items which were culturally appropriate for Uganda.

### Laboratory testing

Participants completed enrollment STI screening, providing blood to test for syphilis via Abbott’s SD Bioline rapid immunochromatographic test (ICT) (Abbott Park, IL) confirmed by rapid plasma reagin (RPR) and first-pass urine specimens to test for chlamydia, gonorrhea, and trichomoniasis via nucleic acid amplification testing (NAAT) with Cepheid’s GeneXpert (Sunnyvale, CA). Not all tests were run the same day due to power instability and study-visit time-constraints.

Participants not previously known to be living with HIV per chart documentation completed enrollment rapid HIV testing with confirmatory testing (using either Abbott’s SD Bioline HIV 1/2 3.0, Abbott’s HIV 1/2 Ag/Ab combo, or Chembio Diagnostic Systems Inc.’s HIV 1/2 Stat-Pak assay (Medford, NY)). All participants completed HIV-RNA testing with Cepheid’s GeneXpert at enrollment and six-months. We defined an undetectable viral load as <400 copies/mL. All laboratory tests were completed at Mbarara University of Science and Technology Research Laboratory.

### STI treatment

Participants with positive STI were treated at the study-site. If STI test results returned after the enrollment visit, participants were called back for treatment. STI treatment regimens followed the Ugandan Ministry of Health Guidelines [25]. Participants with positive STI screening received partner notification card(s) to dispense to partner(s), indicating partner STI exposure and the need for medical evaluation. Participants with infections that could be treated with oral medications (all except syphilis) were offered patient-delivered partner medications.

### Measures

The primary outcome was a positive laboratory test for at least one enrollment STI including syphilis, chlamydia, gonorrhea, and trichomoniasis. Pertinent covariates included age, income, education, number of sexual partners, STI history, intimate and sexual partner violence [15], and sexual relationship power [16, 17]. STI symptoms were not assessed.

### Statistical analysis

We used standard descriptive statistics to summarize and compare the distribution of STI prevalence by covariates with 95% confidence intervals (CI). Bivariable associations between the prevalence of enrollment STIs by enrollment covariates were assessed using the Fisher’s exact test, Wilcoxon rank sum test, and t-test. A p-value < 0.05 was considered statistically significant. Multivariable modeling was not pursued given the small sample size.

### Ethics

Participants provided voluntary, written, informed consent for study participation. Ethical approval was granted by the research ethics boards of Simon Fraser University, Mbarara University of Science and Technology, and Partners Healthcare. Administrative approvals were secured from the Uganda National Council for Science and Technology and the Research Secretariat in the Office of the President.

## Results

### Baseline characteristics

Fifty of the 52 (96%) men meeting enrollment criteria enrolled in the study. Median age was 36.5 (IQR 32–43) years, and 47/50 (94%) were employed (part-time, full-time, or self-employed). Almost all 49/50 (98%) participants were married or living as married with their primary partner, 13/50 (26%) had two or more sexual partners in the prior three months, and 23/50 (46%) reported a history of prior STI. Additionally, 46/50 (92%) had enrollment laboratory-confirmed viral loads < 400 copies/mL (Table 1).

**Table 1:** Baseline socio-demographic characteristics of men living with HIV seeking safer conception care, by STI status (n = 50).

	Total, N = 50 Median (IQR) N (%)	Men with STI, N = 11 Median (IQR) N (%)	Men without STI, N = 39 Median (IQR) N (%)	p-value (unadjusted)^a^
**Sociodemographics**				
Age–median (25–75%)	36.5 (32–43)	33 (31–37)	37 (32–43)	0.27
Education (no school or primary school only)	25 (50%)	5 (45.5%)	20 (51.3%)	1.00
Employed (part-time, full-time, or self-employed)	47 (94%)	11 (100%)	36 (92.3%)	1.00
Household monthly income UGX–median (25–75%)	200,000 (120,000–500,000)	130,000 (100,000–500,000)	200,000 (150,000–500,000)	0.48
Married or living as married with primary partner	49 (98%)	11 (100%)	38 (97.4%)	1.00
Partner age–median (25–75%)	28 (24–30)	25 (22–28)	28 (24–34)	0.31
Alcohol use in the past year	33 (66%)	9 (81.8%)	24 (61.5%)	0.29
Drug use in lifetime	10 (20%)	3 (27.3%)	7 (18%)	0.67
**Sexual and HIV history**				
HIV-RNA viral load				0.56
<400 copies/mL	46 (92%)	11 (100%)	35 (89.7%)	
≥400 copies/mL	4 (8%)	0 (0%)	4 (10.3%)	
Disclosed HIV status to primary partner	46 (92%)	11 (100%)	35 (90%)	0.56
Participant perception of partner with high HIV acquisition risk	16 (32%)	4 (36.4%)	12 (30.8%)	0.73
Prior couples-based HIV counseling and testing	23 (46%)	8 (72.7%)	15 (38.5%)	0.08
≥2 sexual partner in the past 3 months	13 (26%)	3 (27.3%)	10 (25.6%)	1.00
Used condoms at last sex	24 (48%)	4 (36.4%)	20 (51.3%)	0.50
History of prior STI	23 (46%)	7 (63.6%)	16 (41%)	0.31
**Gender and relationship dynamics**				
Sexual and Relationship Power Scale–mean (SD)^b^, [17]	2.45 (0.33)	2.43 (0.25)	2.45 (0.35)	0.90
Sexual and Relationship Power Scale, Decision-Making Dominance Subscale–mean (SD)^,^^b^^,^ [16]	2.32 (0.31)	2.42 (0.39)	2.29 (0.29)	0.22
Dyadic Trust Scale–mean (SD)^c^^,^ [18]	4.37 (0.81)	4.30 (0.91)	4.39 (0.79)	0.73
Slapped or thrown something at partner in prior 12 months	9 (18%)	2 (18%)	7 (18%)	1.00
Forced partner to have sex in past 12 months	6 (12%)	1 (9%)	5 (13%)	1.00
**Used threats or intimidation to coerce partner to have sex in past 12 months**	**4 (8%)**	**3 (27%)**	**1 (3%)**	**0.03**
Social Support–mean (SD)^d^^,^ [19]	2.36 (0.57)	2.21 (0.46)	2.41 (0.60)	0.31

Notes: Bold face indicates statistical significance.

^a^p-values estimated using Fisher’s exact test, Wilcoxon rank sum test, and t-test.

^b^Items are coded so that higher mean indicates higher equity scores.

^c^Items are coded so that higher mean indicates greater trust.

^d^Items are coded so that higher mean indicates greater social support.

### STI prevalence and treatment

**Fig 1:**
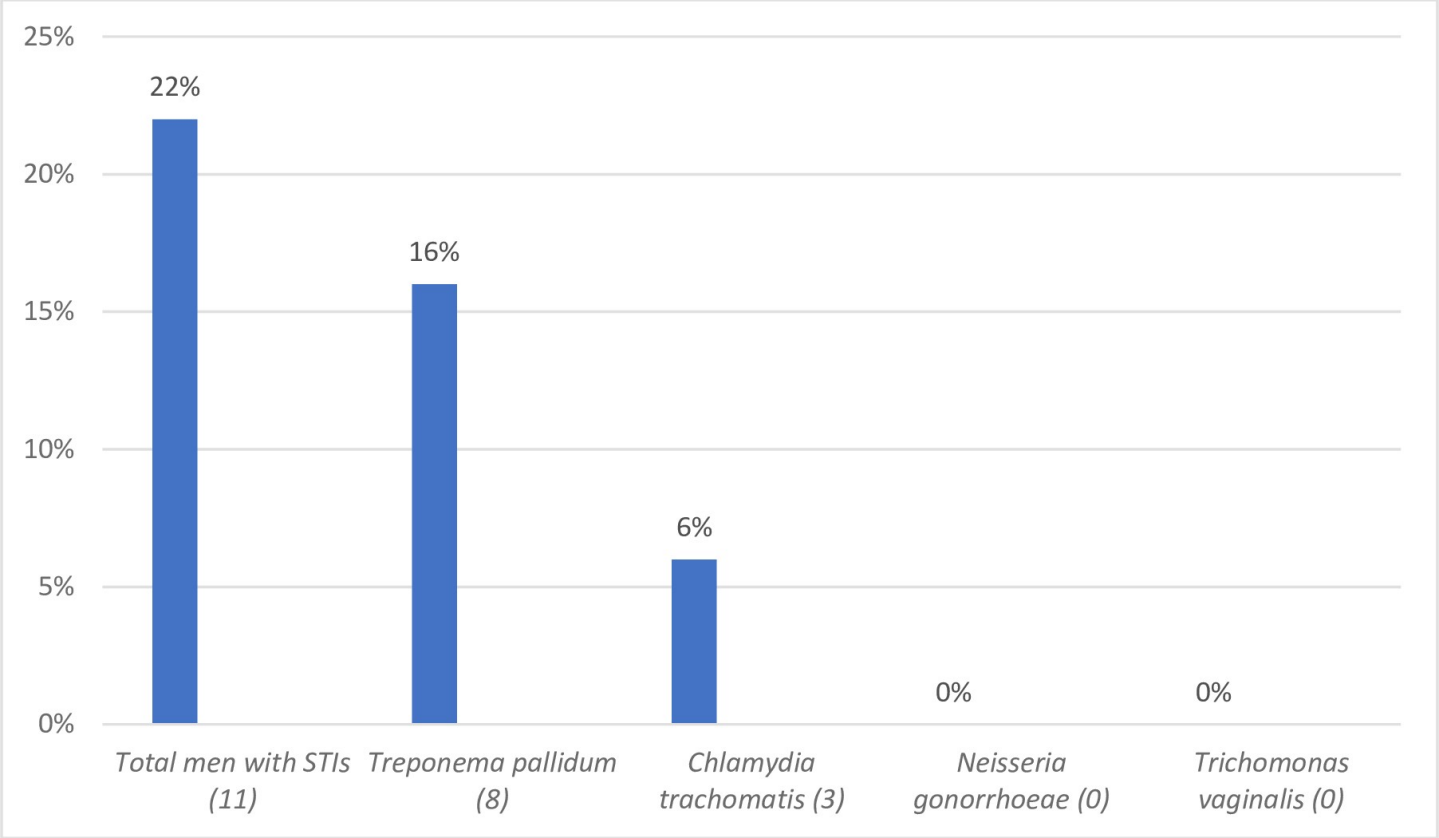
STI prevalence among 50 men living with HIV and seeking safer conception care.

At enrollment, 11/50 (22%) men had at least one STI, including 8/50 (16%) with active syphilis. An additional six participants had positive ICT treponemal antibody testing, but negative RPR testing indicating past infection. Three (6%) had chlamydia, and no participants had gonorrhea or trichomoniasis (Fig 1). Overall, 9/11 participants completed STI treatment. Two participants began syphilis treatment but did not return for subsequent doses (three total required).

### Bivariable comparisons between participants with and without STI

Participants with STI were more likely than those without STI to report using threats or intimidation to coerce partners into sex (127% and 3% respectively; p = 0.03). Other non-statistically significant differences between participants with and without STI are shown in Table 1. Specifically, men with STI were younger and had less social support; more men also had prior STI and reported participating in prior couples-based HIV testing and counseling compared to participants without STI.

## Discussion

We describe a high STI prevalence (22%) among MLWH planning to conceive a child with an HIV sero-different, desired pregnancy-partner. Given that all men enrolled in this study were planning for pregnancy, our data highlight the importance of prioritizing STI screening and treatment for women and men, prior to (in addition to during) pregnancy as a part of HIV prevention and comprehensive SRH.

Our data demonstrate a higher syphilis prevalence compared to other MLWH cohorts. There is a well-described synergy between HIV and syphilis, leading to a higher prevalence of syphilis among populations living with HIV [26–28]. Most data on syphilis prevalence among MLWH focuses on gay, bisexual, and men who have sex with men (gbMSM) with reports of syphilis prevalence ranging from 6–11% across Canada, China, and Uganda [29–31]. A 2011 Ugandan study assessing STI prevalence among key populations of men and women living with HIV (i.e., female sex workers, fishermen, gbMSM), described an 8.5% syphilis prevalence [32]. Strikingly, we found a 16% syphilis prevalence among MLWH who have sex with women, and who would otherwise not be considered to be at higher STI acquisition risk nor undergo routine, STI laboratory screening. Our high syphilis prevalence could be partly due to our use of the reverse algorithm, whereby treponemal testing is followed by non-treponemal testing, resulting in fewer missed diagnoses [33].

We also found a higher chlamydia prevalence (6%) than previously reported in studies of MLWH using NAAT testing, though we found no cases of gonorrhea or trichomoniasis. A cross-sectional analysis of serodifferent couples across Eastern and Southern Africa described a 0.6% 0.9%, and 2.8% prevalence for chlamydia, gonorrhea, and trichomoniasis, respectively, among MLWH partnered with HIV-uninfected women [34]. Additionally, among a cohort of gbMSM in Kampala, Uganda, 1% had urethral chlamydia, 1.1% had rectal chlamydia, 1.4% had urethral gonorrhea, and 1.8% had rectal gonorrhea [29]. Comparing chlamydia and gonorrhea prevalence among gbMSM and men who have sex with women is complicated by gbMSM screening recommendations including pharyngeal and rectal sites in addition to urethral sites, thus capturing more STIs [35]. Our high STI prevalence might be explained by small sample size (i.e., random chance), higher syphilis and chlamydia regional prevalence as evidenced by these pathogens’ high prevalence in HIV-uninfected women from the same site [7], and men increasing STI exposure to meet reproductive goals [36]. Additionally, our lack of gonorrhea or trichomoniasis could be related to circulating antimicrobials and men receiving prior treatment for symptomatic infection.

We found STIs to be significantly associated with sexual coercion, though the absolute numbers of men reporting this was small. This finding, however, is consistent with prior work demonstrating an association between verbal sexual coercion and sexual risk behavior among US women [37]. Research on men has found associations between physical and sexual intimate partner violence and STIs across the world including the US, South Africa, and India [38], though the link between verbal sexual coercion and STIs among men is lacking. More research is needed to understand the connection between verbal sexual coercion and sexual behavior. We anticipate that integrating screening for verbal threats of sexual coercion and violence among women and men into SRH will be integral to identifying individuals vulnerable to STIs as well as violence.

Given Uganda’s high fertility rate [5], men partnered with females of reproductive age spend several years in “periconception periods”. Most STIs are asymptomatic, and without routine STI screening many MLWH and their female partners can contract STIs that remain undiagnosed, untreated, and potentially perinatally transmitted. Most STI research has focused on women, placing the onus of SRH on only half the population. We advocate that including men in SRH will improve the care of men, women, and their families.

For PLWH, HIV clinics typically provide general medical care in addition to HIV services [39] and are a prime setting in which to expand SRH services inclusive of STI screening [40]. Many men otherwise lack regular healthcare interaction. Despite nearly all MLWH in our cohort having undetectable HIV-RNA and disclosing HIV status to their primary partners, 22% had an STI. While HIV prevention and Undetectable (viral load) = Untransmittable U = U [41, 42] is a cornerstone of serodifferent couple counseling (though not yet universally practiced), other aspects of SRH, including STI management, should also be prioritized, especially in the context of safer conception care. To decrease STI transmission and support cure, this additional STI care should focus not only on patients, but also on partners.

### Strengths and limitations

This is a small study, but it is one of the first to demonstrate laboratory-screened STI prevalence among MLWH planning for pregnancy with a female partner.

## Conclusions

Our findings demonstrate a high STI prevalence in this population with increased vulnerability for STI morbidity and transmission to partners and neonates. Future studies are needed to better understand and implement STI screening opportunities for men within HIV care. PLWH generally have several, yearly, healthcare interactions providing opportunities for SRH services inclusive of STI screening and partner outreach. Though men are not typically prioritized for STI screening, their health affects female sexual partners and neonates in addition to themselves; encouraging male engagement in SRH is key to curbing the HIV epidemic.
